# Randomised controlled trial of breast cancer and multiple disease prevention weight loss programmes vs written advice amongst women attending a breast cancer family history clinic

**DOI:** 10.1038/s41416-023-02207-z

**Published:** 2023-02-25

**Authors:** Michelle Harvie, David P. French, Mary Pegington, Cheryl Lombardelli, Suzy Krizak, Katharine Sellers, Emma Barrett, D. Gareth Evans, Ramsey Cutress, Andrea Wilding RGN, Lee Graves, Anthony Howell

**Affiliations:** 1 The Prevent Breast Cancer Research Unit, The Nightingale Centre, Manchester University NHS Foundation Trust, Manchester, M23 9LT England; 2 NIHR Manchester Biomedical Research Centre, Manchester Academic Health Science Centre, Central Manchester University Hospitals NHS Foundation Trust, Manchester, England; 3 Manchester Breast Centre, Oglesby Cancer Research Centre, The Christie, University of Manchester, 555 Wilmslow Rd, Manchester, M20 4GJ England; 4 Division of Cancer Sciences, The University of Manchester, Wilmslow Road, Manchester, M20 4BX England; 5 Manchester Centre for Health Psychology, School of Health Sciences, University of Manchester, Coupland Street, Manchester, M13 9PL England; 6 Department of Medical Statistics, Education and Research Centre, Manchester University NHS Foundation Trust, Manchester, M23 9LT England; 7 Genomic Medicine, Division of Evolution and Genomic Sciences, The University of Manchester, St Mary’s Hospital, Manchester University NHS Foundation Trust, Oxford Road, Manchester, M13 9WL England; 8 University of Southampton and University Hospital Southampton NHS Foundation Trust, Somers Cancer Research Building, Southampton General Hospital, Mailpoint 824, Tremona Road, Southampton, SO16 6YD England; 9 Tameside Macmillan Unit/Breast Service, Tameside and Glossop Integrated Care NHS Foundation Trust Fountain Street, Ashton-under-Lyne, OL6 9RW UK; 10 School of Sport and Exercise Sciences, Liverpool John Moores University, Liverpool, L3 5UX England; 11 Department of Medical Oncology, The Christie NHS Foundation Trust, Wilmslow Rd, Manchester, M20 4BX England

**Keywords:** Weight management, Nutrition

## Abstract

**Background:**

Overweight and obesity are common amongst women attending breast cancer Family History, Risk and Prevention Clinics (FHRPCs). Overweight increases risk of breast cancer (BC) and conditions including^1^ cardiovascular disease (CVD) and type-2 diabetes (T2D). Clinics provide written health behaviour advice with is likely to have minimal effects. We assessed efficacy of two remotely delivered weight loss programmes vs. written advice.

**Method:**

210 women with overweight or obesity attending three UK FHRPCs were randomised to either a BC prevention programme (BCPP) framed to reduce risk of BC (*n* = 86), a multiple disease prevention programme (MDPP) framed to reduce risk of BC, CVD and T2D (*n* = 87), or written advice (*n* = 37). Change in weight and health behaviours were assessed at 12-months.

**Results:**

Weight loss at 12 months was −6.3% (−8.2, −4.5) in BCPP, −6.0% (−7.9, −4.2) in MDPP and −3.3% (−6.2, −0.5) in the written group (*p* = 0.451 across groups). The percentage losing ≥10% weight in these groups were respectively 34%, 23% and 14% (*p* = 0.038 across groups).

**Discussion:**

BCPP and MDPP programmes resulted in more women achieving ≥10% weight loss, but no evidence of additional benefits of MDPP. A multicentre RCT to test the BCPP across UK FHRPCs is warranted.

Clinical Trial Registration ISRCTN16431108.

## Introduction

Observational data suggest weight control, physical activity (PA), healthy diet, alcohol and smoking limitation reduce breast cancer (BC) risk in women at increased risk of the disease [1–5]. Optimising weight and health behaviours will reduce risk of BC, other cancers and other conditions including cardiovascular disease (CVD), type 2 diabetes (T2D) and dementia. Unhealthy behaviours, overweight and obesity are common amongst increased-risk women attending Family History, Risk and Prevention Clinics (FHRPCs) [6, 7]. Current UK familial BC guidelines recommend that women should be advised on the increased risks of overweight/obesity, sedentariness, alcohol and smoking [8]. Current standard care involves general written advice which is likely to have a minimal effect on health behaviours [9]. Thus, current approaches are unlikely to adequately manage risk in the FHRPCs and are missing an opportunity to prevent BC and other diseases.

The optimal weight loss programmes for women at increased risk are not currently known. In the study reported here we assess remotely delivered weight reduction programmes likely to be preferable to clinic attendees who often live far from the specialist FHRPCs. Remote programmes could also be delivered across a network of FHRPCs from a central centre by health care professionals with appropriate training and skills, thus avoiding the need for local delivery teams, as utilised in the ongoing Breast Cancer Weight Loss (BWEL) trial in the US and Canada [10].

Women in the FHRCP can have increased risk markers for CVD and T2D [7] and there may be overlap between risk markers and risk for CVD, T2D and BC [11, 12]. Women at high risk of BC in the FHRPC often have a belief that improving health behaviour can reduce their risk of CVD to a greater extent than their risk of BC (greater response efficacy), as BC risk is perceived to be under genetic control [13, 14]. This study is testing the hypothesis that additional personalised CVD and T2D risk information increases the probability of engagement and adherence to the weight loss programme compared to just receiving information on their risk of BC. Additional personalised CVD and T2D risk could enhance weight loss success due to a greater response efficacy for CVD and T2D compared to the risk of BC [13, 14]. However CVD and T2D risk could attenuate weight loss success as this risk information may be less personally relevant to women at increased risk of BC which could decrease engagement [15].

This trial aimed to identify whether the remote programmes performed better than written advice to engage women in the FHRPC to lose weight. Also, whether a Breast Cancer Prevention Programme (BCPP) or a multiple disease prevention programme (MDPP) performed best and could be tested in a future definitive trial to identify the clinical and cost effectiveness of the relevant intervention across the UK FHRPC network. The primary outcome of the trial was percentage weight loss at 12 months since long term weight loss is considered most relevant for cancer prevention. Secondary outcomes included the numbers with greater than or equal to 5 and 10% weight loss in the groups, retention to the trial, changes in body composition, health behaviours and fidelity of delivery of the programmes. Process analysis of the trial (both qualitative and quantitive), health economic analyses, and changes in breast density with weight loss will be reported elsewhere.

## Materials and methods

### Study design

We conducted a multi-centre prospective three arm randomised controlled trial of written advice vs BCPP vs MDPP amongst women attending FHRPCs at Manchester University NHS Foundation Trust (MFT), Tameside and Glossop Integrated Care NHS Foundation Trust (T&GICFT) and the University Hospital Southampton NHS Foundation Trust (UHS).

### Participants

We included women previously identified according to NICE guidelines [8] as being at either moderate (≥17% to 29.99%) or high (>30%) lifetime risk of BC, aged ≥30 years with overweight or obesity (BMI ≥ 25 kg/m^2^). Previous personalised estimates of BC risk had been derived using the Tyrer-Cuzick model (version 8), which includes family history, hormonal risk factors, BMI+/− visually assessed mammographic density (Breast Imaging Reporting and Data System, BI-RADS) and a polygenic risk score (SNP 18) if these were available [16] in MFT and T&GICFT or had been based solely on family history information according to NICE CG164 guidance in UHS. Women were excluded if they did not have access to a phone or the internet, had a previous diagnosis of cancer, T2D or CVD, were currently prescribed statins, had a major physical or psychiatric condition which made them unsuitable for a home based diet and physical activity programme, were receiving weight loss medication (Orlistat), had previous bariatric surgery, or were already successfully following a diet and/or physical activity plan and had lost more than 1 kg of weight in the last 2 weeks. Only one woman per family was able to join the trial to avoid contamination between the groups.

### Procedure

#### Recruitment

Women were invited by both postal letter and face-to-face during appointments at the recruiting centres by the clinic nurses/clinicians/radiographers. Interested women were asked to check their eligibility on the trial website or by phoning the trial office. The invitation letter included an opt-out slip to indicate reasons they were not eligible or not interested.

#### Randomisation and stratification

Randomisation was undertaken using a minimisation programme located on a computer in each of the recruiting centres by a trial administrator not involved in delivery of the programmes. Randomisation was stratified by four factors:

1. BMI < or ≥ projected median of 30 kg/m^2^
2. Age < or ≥ projected median of 45 years in the FHRPC since weight loss success is often greater amongst older participants
3. Moderate or high risk of BC as calculated by the local FHRPC (i.e. lifetime Tyrer- Cuzick risk ≥17–29.99% or >30%)
4. Recruiting centre

The study involved unequal randomisation to the three groups to allow the most efficient design to consider the larger predicted clinically important weight difference between the minimal intervention written group and each of the two programmes, as well as the smaller expected difference between the two different programmes. With 30 in the minimal intervention group and 74 subjects in each of the two ‘active’ treatment groups, the trial had 90% power to detect differences in percentage weight loss of 4% or more between the control group and an active treatment group, and a difference of 3% or more between the two active treatment groups. The study was powered for these two analyses as reported previously [17]. Incorporating an estimated drop-out rate of 15%, these sample sizes increase to 35 controls and 86 in each of the two active treatment groups. These calculations are based on the two-tailed independent-groups t-test with estimated SD of 5%, and with a 2% significance level to account for multiple testing between the three groups.

#### Interventions: Written advice, BCPP and MDPP programmes

The weight loss programmes were delivered remotely by dietitians at MFT.

**Fig. 1:**
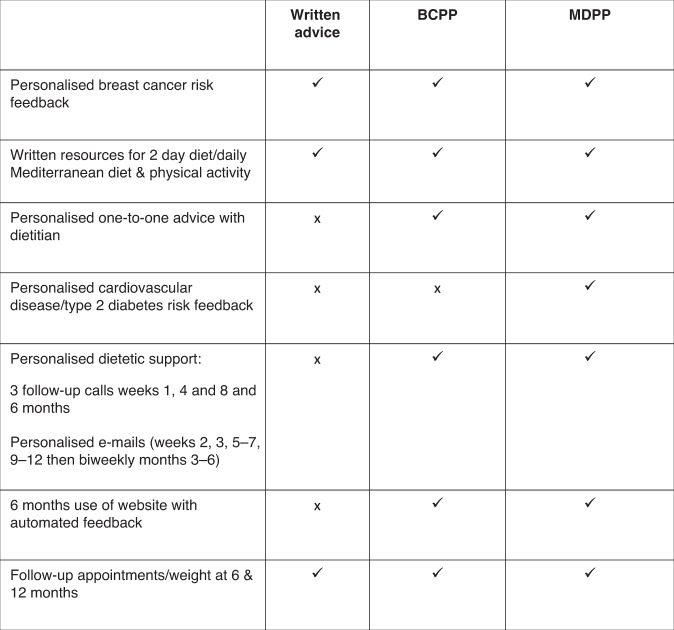
Components of the BCPP, MDPP and written advice.

The main components of the programmes are described in Fig. 1, with more detailed information in Supplementary Fig. 1. Some women had their initial BC risk estimated a number of years previously. Therefore, participants in all groups had their personalised risk of BC re-estimated at the start of the trial to ensure breast cancer risk was contemporaneous to the additional CVD and T2D risks. Breast Cancer risk was communicated by a clinician in their recruiting FHRPC during a phone or face to face consultation along with verbal advice on how risk could be reduced through weight loss of 5–10% and health behaviour change. All groups received identical comprehensive written information to follow either a weight reducing intermittent (5:2) or daily energy restricted Mediterranean diet including portion guides and recipes as described previously [18]. They also received a detailed booklet which outlined the benefits of physical activity and a home based programme designed to meet physical activity recommendations (150 min of moderate intensity cardiovascular and 40 min of resistance exercise/week) [19, 20]. All groups received a monthly trial newsletter.

The BCPP group just received their personalised BC risk information. The MDPP group had an NHS Health Check at their baseline appointment which included point of care testing and feedback of their total and HDL cholesterol and HbA1c (Afinion Abbott UK), and personalised risk feedback for developing CVD (10-year and lifetime risk and heart age from QRISK2 [21]) and T2D (10-year risk from QDiabetes [22]).

The BCPP and MDPP programmes both included ongoing remote support from a dietitian trained in disease risk communication and prevention of BC, CVD, T2D and dementia through health behaviour change. Both programmes were supported by a trial website which included self-monitoring of weight, diet (completion of restricted days in the 5:2 diet and actual food and drink intake), physical activity (both cardiovascular and resistance), and an average weight loss line for their group to allow social comparison. There were separate moderated forums for BCPP and MDPP to avoid contamination between the groups, where women could message other participants and ask questions of the trial dietitians. Also, weekly menu plans, recipes, tips for planning and managing emotional eating, online videos of the recommended resistance exercises (Physiotec, Canada) and general information about BC, CVD, T2D and dementia and the prevention of these conditions.

Women received tailored feedback on their self-reported behaviours on the website from their allocated trial dietitian in the first 6 months. Months 0–3; scheduled phone calls weeks 1, 4, 8 and personalised e-mails in weeks 2, 3, 5–7 and 9–12. Months 3–6; personalised emails every two weeks. Between 6-and 12-months women received automated monthly emails in response to website entries.

The intervention components were consistent with the Health Action Process Approach a widely-used model of behaviour change that distinguishes between different stages of behaviour change [23]. First, both programmes provided disease risk information as a gain framed message [24]. This was augmented by providing information about how weight loss would reduce disease risk (response efficacy) and promoting mastery experience of successfully performing key behaviours to lose weight, thereby increasing self-efficacy. The programmes also included behaviour change techniques that promoted self-regulation of behaviour, including goal setting, planning, self-monitoring, and encouraging individuals to identify sources of social support for changing behaviours [25]. The use of intermittent dieting was also intended to help with relapse prevention [26] and use of prompts were employed to make the behaviour habitual [27]. Consideration of emotional eating was an important part of the intervention that is not well captured by the Health Action Process Approach [28].

### Measures

Trial outcomes were assessed at baseline, 6 and 12 months at face-to-face appointments in the recruiting centres. Trial assessments were undertaken by research assistants who were not delivering the programmes to attempt to ensure the assessor was blind to the allocation group. However, participants were aware of their allocation group and were likely to communicate this during the assessment.

#### Primary outcome: Percentage weight loss

Weight was assessed using weight and body composition analysers in the three centres (MFT Tanita 180, T&GICFT Tanita 420, UHS Seca multi-frequency mBCA 515). Measurements at the different time points were conducted on the same machines for each trial participant.

#### Secondary outcomes

Body fat and fat free mass (bioelectrical impedance), waist and hip circumference were assessed using standardised methods as described previously [18]. Health behaviours were assessed using validated questionnaires; diet quality (12-point Mediterranean diet score) [29], physical activity (IPAQ short form) [30], alcohol (7-day recall [31] AUDIT alcohol use disorders test) [32] smoking behaviour (never/ex-smoker or current smoker, number of cigarettes/day). Resting blood pressure was assessed at baseline in the three groups to determine the safety of participants to undertake physical activity and reassessed at 12 months. Patients in the BCPP and written advice groups did not see their blood pressure measurements when they were taken and were only informed of adverse results which required further investigations by their GP.

#### Additional baseline assessments

These included weight and dieting history, and scales for anxiety (Generalised Anxiety Disorder GAD [33]) depression (Patient Health Questionnaire PHQ [34]) and binge eating [35].

#### Fidelity of delivery and engagement with BCPP and MDPP

We assessed the numbers of scheduled calls and e-mails received, engagement with the web site and the amount of dietitian time used to deliver the programmes, also the number of women referred to NHS behaviour change services (i.e. exercise on referral, alcohol and smoking cessation services).

### Adverse events

The number of serious or unexpected adverse events were recorded throughout the 12-month trial period.

### Analysis

#### Statistical analysis

Summary statistics are presented as mean (SD) or median (IQR) for continuous variables, and as numbers and percentages for categorical variables. Percentage weight loss at 6 and 12 months was compared between the three treatment groups, using analysis of covariance (ANCOVA) with baseline weight included as a covariate. Additional adjustments were made for a priori baseline confounders; estimated lifetime BC risk, age, Townsend deprivation score, anxiety and depression scores. Adjusted means and 95% confidence intervals (CIs) are reported, along with global F-tests to compare differences between the groups, and post-hoc pairwise comparisons where appropriate. Multiple imputation was used for missing outcome data at 6-month and 12-month time points, using baseline data and 6-month outcome data if available. Predictive mean matching was used for the imputation with the ‘mice’ package for R statistical software [36]. The proportion of patients achieving 5% and 10% weight loss at 12-months is also presented for each treatment group and compared using Chi-square test and pairwise proportion tests for intervention groups versus control group with Benjamini-Hochberg adjustment for multiple testing.

A longitudinal analysis, using generalised estimating equations (GEE), was used to assess differences between the groups in percentage weight loss over the 6-month and 12-month time points adjusting for baseline weight and a priori confounders. Secondary analyses compared changes in body composition, blood pressure and health behaviours (Mediterranean diet score, physical activity, alcohol intake and smoking behaviour) at 12-months, using ANCOVA and adjusting for baseline measurements.

The main analyses were on an intention-to-treat basis using R version 4.0.2 at the 5% significance level unless otherwise stated. Sensitivity analyses checked the missing at random assumption by comparing treatment effects for completers only and a baseline observation carried forward analysis, and explored differences in baseline characteristics between women who completed vs those who did not complete the trial.

## Results

### Recruitment, retention and baseline patient characteristics

**Fig. 2:**
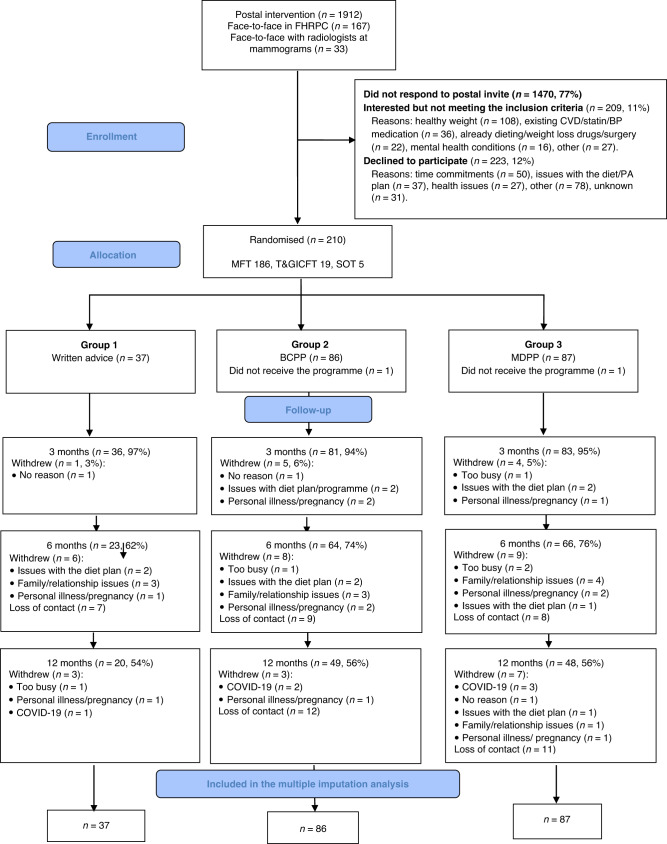
Consolidated Standards of Reporting Trials (CONSORT) flow diagram of patients recruited to the trial.

Recruitment was between December 2017 and March 2019This period was a median (minimum, maximum) 5.0 (0–27.7) years after women had joined the FHRPC. We recruited 37 to written advice, 86 to the BCPP and 87 to the MDPP. Overall uptake was 10% (210/2112) of those invited (7% [126 out of 1912] by post, 42% [84/200] in-person invitation) (Fig. 2). Recruitment was 89% (186/210) from MFT. Table 1 shows women were primarily white (95%), non-smokers (95%) with a range of deprivation scores and a relatively high prevalence of physical and psychological co-morbidities. After receiving updated BC risk information at the start of the trial around half of women remained in their original risk category, 31% decreased a risk category and 9% increased a risk category. Approximately a quarter of women had a family history of CVD or T2D. Twelve percent of the cohort had opted to take BC risk reducing medication. Many of the women had multiple previous attempts to lose weight, median (IQR) 4 (2–9), and 71% had previously attended commercial weight loss programmes.

**Table 1:** Baseline characteristics.

	Written advice (*n* = 37)	BCPP (*n* = 86)	MDPP (*n* = 87)
Study centre *n* (%)			
MFT	32 (87%)	75 (88%)	79 (91%)
T&GICFT	3 (8%)	9 (10%)	7 (8%)
UHS	2 (5%)	2 (2%)	1 (1%)
Age median (IQR)	49 (44–52)	48 (44–52)	48 (43–51)
Pre/postmenopausal *n* (%)	24/13 (65/35%)	55/31 (64/36%)	51/36 (58/42%)
Ethnicity *n* (%)			
White	34 (92%)	82 (95%)	84 (96%)
Asian/Black	3 (8%)	4 (5%)	3 (4%)
BMI (kg/m^2^) median (IQR)	30.4 (28.3–32.8)	31.1 (28.4–36.1)	31.6 (27.8–36.2)
Overweight (BMI 25–29.99 kg/m^2^) *n* (%)	17 (46%)	37 (42%)	38 (44%)
Obese (BMI > 30 kg/m^2^) *n* (%)	20 (54%)	49 (58%)	49 (56%)
Smoking status *n* (%)			
Current	3 (8%)	4 (5%)	5 (6%)
Previous	13 (35%)	29 (33%)	30 (34%)
Never	21 (57%)	53 (62%)	52 (60%)
Years since joining the FHRPC median (IQR)	5.5 (2.9–10.3)	4.1 (1.0–10.8)	5.7 (2.3–12.4)
Estimated % lifetime BC risk at baseline (T-C) mean (SD)	26 (9)	25 (10)	24 (12)
Moderate (≥17–29.99%)	26 (70%)	66 (77%)	68 (78%)
High (>30%)	11 (30%)	20 (23%)	19 (22%)
Change in breast cancer risk category between joining the FHRPC and the study			
Increase	4 (11%)	7 (8%)	7 (8%)
Stable	26 (70%)	43 (50%)	46 (53%)
Decrease	7 (19%)	30 (34%)	28 (32%)
Not updated	0 (0%)	7 (8%)	5 (6%)
Gene variant status			
Positive individual	1 (3%)	0 (0%)	3 (3%)
Positive family & individual untested	0 (0%)	1 (1%)	3 (3%)
Positive family & individual negative	0 (0%)	3 (3%)	2 (2%)
Prescribed BC risk reducing medication			
Already taking at baseline	1 (3%)	5 (6%)	3 (3%)
Commenced at baseline	4 (11%)	10 (11%)	2 (2%)
Family history of CVD^a^	9 (24%)	22 (26%)	17 (20%)
Family history of T2D^b^	10 (27%)	18 (17%)	31 (36%)
IMD Quintile			
1 (most deprived	6 (16%)	13 (15%)	20 (23%)
2	8 (21%)	12 (4%)	12 (14%)
3	8 (22%)	16 (18%)	23 (26%)
4	8 (22%	17 (20%)	15 (17%)
5 (least deprived)	7 (9%)	28 (33%)	17(20%)
Anxiety (PHQ) median (IQR)	3 (2–7)	3 (1–5)	3.5 (1–6.8)
Moderate/severe *n* (%)	3 (9%)	5 (6%)	10 (12%)
Missing	*N* = 1	*N* = 3	*N* = 3
Depression (GAD) (*n* = 202)			
Median (IQR)	4 (3–10)	3 (1.5–6)	4 (2–7)
Moderate/severe *n* (%)	8 (23%)	13 (15%)	10 (12%)
Binge eating score 4	4 (11%)	8 (10%)	13 (17%)
Missing	*N* = 1	*N* = 3	*N* = 3
Living situation *n* (%)			
Lives with partner	31 (84%)	67 (78%)	69 (79%)
Lives alone	3 (8%)	14 (16%)	17 (20%)
Lives in shared house	3 (8%)	5 (6%)	1 (1%)
Dependent children at home	25 (69%)	60 (73%)	59 (69%)
Highest education level *n* (%)			
Primary & secondary	4 (11%)	15 (17%)	18 (21%)
Further	16 (43%)	22 (26%)	19 (22%)
Higher	16 (43%)	46 (57%)	47 (54%)
Missing	1 (3%)	3 (4%)	3 (3%)
Employment status *n* (%)			
Full time	22 (60%)	61 (71%)	60 (69%)
Part time	7 (20%)	19 (22%)	23 (27%)
Unemployed/long term sick	4(10%)	2 (2%)	0 (0%)
Full time student	2 (5%)	2 (2%)	0 (0%)
Home carer	0 (0%)	2 (2%)	2 (2%)
Retired	2 (5%)	0 (0%)	2 (2%)
Any chronic health condition	29 (78%)	58 (68%)	60 (69%)
Anxiety/depression	5 (14%)	9 (10%)	8 (9%)
Musculoskeletal	9 (24%)	15 (17%)	17 (20%)
Asthma	2 (5%)	12 (14%)	5 (6%)
Gastrointestinal	2 (5%)	10 (12%)	8 (9%)
Thyroid	2 (5%)	5 (6%)	4 (5%)
Number of previous weight loss attempts			
n (IQR)	3 (2–6)	4 (2–9)	4 (3–10)
≤2	12 (33%)	25 (29%)	20 (21%)
3–4	9 (24%)	21 (24%)	30 (35%)
5–9	4 (11%)	20 (23%)	14 (21%)
>9	12(33%)	21 (24%)	17 (20%)
Previously advised to lose weight by HCP	16 (43%)	42 (49%)	45 (52%)
Previous NHS weight loss programme	1 (3%)	9 (11%)	6 (7%)
Previous commercial weight loss programme	24 (65%)	61 (71%)	65 (75%)

^a^Family history of CVD: first degree relative who has had stroke, transient ischaemic attack, myocardial infarction, angina or peripheral vascular disease <age 60 years.

^b^Family history of T2D: First degree relative with T2D at any age.

Seven BCPP (8%) and five MDPP (6%) did not receive updated BC risk information. One BCPP and one MDPP did not have their initial personalised diet and risk information as they did not engage with the programme after recruitment (Fig. 2). Retention was 56% overall and was comparable between the three groups. Retention was higher at MFT (110 out of 186; 59%) compared to T&GICFT (5 out of 19; 26%) and UHS (2 out of 5; 40%). The majority of drop out in the written advice group occurred in the first 6 months (14/17; 82%), whilst many participants in the BCPP (15/37; 41%) and the MDPP groups (18/39; 46%) left the trial between 6 and 12 months during the web and automated feedback phase. Across all groups, women who left the trial were on average two years younger, had a higher deprivation score and baseline BMI (Supplementary Table 1). Neither the initial nor updated BC risk scores were associated with withdrawal.

Eight women were still active in the trial at the start of the COVID-19 pandemic in March 2020. Five patients withdrew (2 BCPP, 3 MDPP), and three had their 12-month appointments by phone and provided self-reported weight (1 written, 2 MDPP).

### Change in weight

**Fig. 3:**
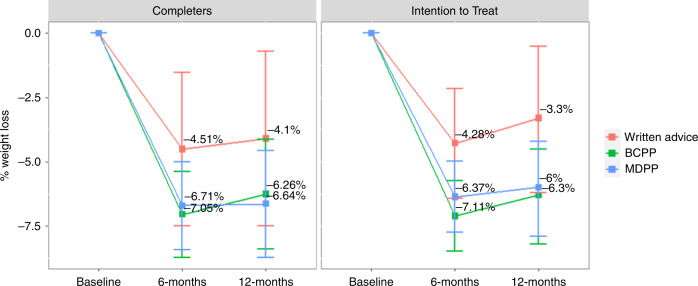
Percentage weight loss in the 3 groups over 12 months ANCOVA estimated marginal means for completers and intention to treat using imputed data.

Percentage change in weight using imputed data is reported in Table 2. Weight reduced in all groups at 6 and 12 months with some weight regain between 6–12 months. Mean (95% CI) percentage weight loss at 12 months was numerically higher in the BCPP −6.3 (−8.2, −4.5) % and the MDPP groups −6.0 (−7.9, −4.2) % compared to the written group −3.3 (−6.2, −0.5) % with wide confidence intervals and no statistically significant difference between the groups (*p* = 0.451). Weight loss of ≥10% was more likely in the BCPP (34%) and the MDPP (23%) group compared to the written group (14%) (*p* = 0.042). Written vs BCPP *p* = 0.053, written vs MDPP *p* = 0.328. Similar results were seen in the per protocol analysis (Table 2, Fig. 3). Also, the baseline observation carried forward analysis; mean (95%CI) weight change BCPP −3.3 (−4.6, −2.0)%, MDPP −3.8 (−5.1, −2.5)% compared to −2.0 (−4.0, 0.0)% in the written group (*p* = 0.323). Weight loss of ≥10% was more likely in the BCPP (20%) and the MDPP (13%) group compared to the written group (3%) (*p* = 0.031), written vs BCPP (*p* = 0.038) written vs. MDPP (*p* = 0.105).

**Table 2:** Percentage change in weight at 6 and 12 months in the three groups using imputed data.

	Baseline – weight (kg)	6 months weight loss (%)	*P* value	12 months weight loss (%)	*P* value	Weight loss categories – 12 months n (%)			*P* value
						<5%	5–10%	>10%^a^	
Written (*n* = 37)	83.6 (11.1)	−4.3 (−6.4, −2.2)	*p* = 0.384	−3.3 (−6.2, −0.5)	*p* = 0.451	18 (49%)	14 (37%)	5 (14%)^b^	^a^*P* = 0.038
BCPP (*n* = 86)	87.2 (15)	−7.1 (−8.5, −5.7)		−6.3 (−8.2, −4.5)		35 (40%)	22 (25%)	30 (34%)^b^	^b^*P* = 0.018
MDPP (*n* = 87)	89.2 (16.7)	−6.4 (−7.8, −5.0)		−6.0 (−7.9, −4.2)		42 (49%)	24 (27%)	20 (23%)	^c^*p* = 0.324

Mean (95% CI) EMM: Estimated marginal means Imputed: m = 50 datasets, 2 outliers were not removed.

Adjusted: Adjusting for baseline weight, updated risk, risk difference, age, deprivation, anxiety/depression.

^a^*p* = 0.038 all groups.

^b^p = 0.062 Written vs BCPP.

^c^*p* = 0.324 Written vs MDPP post-hoc comparisons which include adjustment for multiple testing.

### Change in secondary end points

#### Body composition, blood pressure and health behaviours

All groups reduced body fat, waist and hip measurements and blood pressure, which were more marked amongst the BCPP and MDPP groups (Table 3).

**Table 3:** Change from baseline in secondary end points in participants who completed the study in the three groups.

	Baseline	Change at 6 m	Change at 12 m	Change at 12 m^a^ EMM (95%CI)	*P* value^b^
Body Fat					0.363
Written	33.2 (7.73)	−2.72 (3.81)	−2.15 (4.62)	−2.72 (−5.72,0.29)	
BCPP	35.0 (10.3)	−4.56 (5.22)	−5.52 (7.56)	−5.27 (−7.2,−3.4)	
MDPP	35.9 (11.6)	−3.97 (4.6)	−4.31 (5.94)	−4.32 (−6.2,−2.5)	
Fat free mass (kg)					0.46
Written	50.4 (4.91)	−0.61 (3.68)	−0.72 (2.55)	−0.98 (−2.44,0.49)	
BCPP	52.1 (6.68)	−2.04 (3.49)	−2.04 (3.4)	−2.05 (−2.9,−1.15)	
MDPP	53.3 (6.65)	−1.92 (2.51)	−1.72 (3.09)	−1.61 (−2.5,−0.7)	
Waist (cm)					0.126
Written	103 (12.0)	−2.91 (5.9)	−3.24 (4.24)	−3.89 (−7,−0.81)	
BCPP	105 (13.0)	−8.66 (14.8)	−7.9 (6.83)	−7.72 (−9.7,−5.7)	
MDPP	106 (13.1)	−4.97 (6.13)	−6.51 (7.51)	−6.41 (−8.4,−4.5)	
Hip (cm)					0.164
Written	113 (8.52)	−9.53 (26.3)	−2.2 (4.3)	−2.54 (−5.6,0.51)	
BCPP	115 (9.53)	−10.7 (24.7)	−6.14 (7.87)	−6.04 (−8,−4.1)	
MDPP	115 (12.1)	−12.9 (29.6)	−5.37 (5.87)	−5.33 (−7.2,−3.4)	
Systolic BP (mm/hg)					0.107
Written	122 (16.6)		−1.91 (11.3)	−2.67 (−7.6,2.2)	
BCPP	127 (19.9)		−8.52 (12.6)	−7.46 (−10.7,−4.3)	
MDPP	127 (14.6)	No data	−8.27 (11.5)	−9 (−12.2,−5.8)	
Diastolic BP (mm/hg)					0.183
Written	78.7 (9.19)		−3.07 (7.6)	−3.7 (−7,−0.43)	
BCPP	80.4 (9.85)		−7.45 (7.5)	−7.18 (−9.3,−5.1)	
MDPP	81.5 (8.11)	No data	−5.33 (7.43)	−5.32 (−7.5,−3.2)	

Mean (SD) Estimated marginal means (95% CI).

^a^Completers EMM adjusting for baseline measurement, breast cancer risk, age, deprivation, anxiety (GAD) and depression (PHQ).

^b^Completers F-test.

All groups showed some improvements in self-reported health behaviours (Supplementary Table 2). Diet quality increased at 6 months and was maintained at 12 months in all groups. Increases in PA at 12 months were modest in the written group and more marked amongst the BCPP and MDPP groups. The median metabolic equivalent MET-minutes/ week reported were respectively equivalent to an additional 12, 36 and 60 min of moderate intensity PA/week. Alcohol reduced in all groups at 3 and 6 months with a slight increase at 12 months. Of the 12 smokers recruited at baseline (3 written, 4 BCPP, 5 MDPP), 5 completed the trial (2 written, 1 BCPP, 2 MDPP). One of the written advice group had stopped smoking and one of the BCPP group had reduced the number of cigarettes smoked.

### Cardiovascular and type 2 diabetes disease risk information

Baseline CVD and T2D risk and risk markers in the MDPP group are reported in Supplementary Table 3. The proportion with sub-optimal risk markers and at increased CVD and T2D risks were as follows; total cholesterol (>5 mmol/L) *n* = 30 (34%), systolic blood pressure (>130 mm/Hg) *n* = 41 (47%), lifetime CVD QRISK2 (>25%) *n* = 26 (30%), QDiabetes 10-year risk (>5.6) *n* = 39 (45%). Completers in the MDPP group (*n* = 45) experienced small reductions in mean (95%) lifetime CVD risk −1.9 (−3.1 to −0.76) %, 10-year QDiabetes risk −1.1 (−1.9 to −0.38) % and HbA1c −1.0 (−1.6 to −0.5) mmol/mol but no change in total cholesterol +0.2 (−0.1 to +0.4) mmol/L.

### Fidelity of programme delivery in the groups

Subjects in the BCPP and MDPP groups received most of the planned dietitian calls and e-mails (Supplementary Table 4a). There was high engagement with the website across both groups during the first 3 months (respectively 94% and 93% of those recruited) and continued usage amongst 81% of the BCPP and 70% of the MDPP who remained in the trial during months 9–12. There was a poor engagement with the peer support forum which was only used by 9% of the BCPP and 15% of the MDPP groups. Twelve percent of women in the BCPP and MDPP groups were referred to NHS exercise on referral services, and a small number were referred for psychological support, alcohol and smoking cessation services. The majority of women opted to follow the intermittent diet at the start of the trial (90% BCPP, 86% MDPP). Only half of completers were still following an intermittent diet (one or two calorie-restricted days/week) to maintain weight loss at 12 months (47% BCPP, 54% MDPP). Small but comparable numbers of completers in the three groups reported accessing other commercial weight loss services during the trial: written 3 (15%), BCPP 5 (10%), and MDPP 7 (15%)(Supplementary Table 4b).

### Adverse events

Two patients experienced serious adverse events and were admitted to hospital with migraine (BCPP *n* = 1) and a leg fracture (MDPP *n* = 1). These were not related to the interventions.

## Discussion

Weight loss occurred in the BCPP and MDPP programmes and the minimal intervention written group, although the supported programmes resulted in more women achieving clinically significant weight loss of ≥10%. Additional personalised CVD and T2D risk information did not influence weight loss success compared to a programme which just provided personalised BC risk.

We have shown good uptake to the trial, particularly from face-to face recruitment. Uptake of 10% with a postal invitation was comparable to previous weight loss studies in our high risk clinic (12%) [37], other high cancer risk populations (13%) [38] and women in the general population with overweight or obesity (8.5%) [39]. Retention at 12 months was just below 60%. This aligns with previous weight loss intervention studies amongst women attending breast screening [18] and women in the general population [40].

Previous randomised controlled trials amongst women at increased risk of BC have involved smaller cohorts (50–80 participants) and reported weight loss at the end of the active weight loss phase at either 3, 4 and 6 months [38, 41, 42]. We have reported the longer-term effects on weight loss maintenance. Some weight was regained in the last 6 months despite continued use of the website by many of the completers. Weight loss success has been linked to ongoing contact and accountability to a human coach rather than self-monitoring and standardised automated feedback as used in our study [43]. Maintenance of weight loss in future programmes could be enhanced by extended health care professional support, use of video calls which may enhance the therapeutic relationship compared to the standard audio calls used herein [44] and specialist behavioural input from a clinical psychologist [45]. Enhanced peer support has the potential to maintain sustained behaviour change. The web forums were poorly utilised which limited their potential efficacy. Peer support could potentially be improved by including some visual and audio communication to foster a sense of community and connectivity between users [46].

Weight loss in our written advice group (3.3%) was considerably higher than the 1% previously reported in the literature. This previously reported 1% weight loss in controls is thought to be an effect of trial weigh-ins [47]. Our minimal intervention group may have been more effective than previous control groups as women were all motivated to lose weight and it also included some elements of the BCPP and MDPP programmes which enhance weight loss and health behaviour change. These include the initial one-to-one counselling on the importance of weight control from the FHRPC clinician [48], and the detailed prescriptive dietary advice with food portion guides [49]. The individualised diet and PA advice and ongoing dietitian behaviour change support achieved the expected increased weight loss in the two intervention groups [50, 51]. It specifically increased the number of women able to lose larger amounts of weight (≥10%).

Modest weight loss of ≥5% has been associated with reduction in risk of postmenopausal BC within observational studies [52, 53]. However recent studies have reported reductions in BC biomarkers with weight loss of ≥10% rather than 5% [54, 55] suggesting the target weight loss for BC risk reduction is 10%. Increasing the numbers with 10% weight loss in future interventions could be achieved by including an intensive low calorie diet period at the start [56] ongoing use of meal replacements [57] or use of emerging effective GLP-1 agonist weight loss medications [58].

There was no difference in retention or weight loss success between the MDPP and BCPP groups which is consistent with our findings in women in the general population attending breast screening [18]. Prior to this trial it was unclear whether additional personalised CVD and T2D risk to women in the FHRPC would enhance or decrease weight loss success due to a greater response efficacy for CVD and T2D compared to the risk of BC [13, 14] or CVD/T2D risk information being less personally relevant to women at increased risk of BC [15].

There was no clear benefit of the additional CVD and T2D risk information. Notably 55–70% of the MDPP group had low estimated 10-year and lifetime risks of CVD and T2D. Low CVD risk factors (i.e., lipid and blood pressure) may lead to underestimation of subsequent lifetime risk amongst the relatively young women in the FHRPC. It is not known whether better lifetime CVD risk assessment tools could increase engagement amongst young women [59].

Actual level of BC risk amongst these women at increased risk did not predict retention to the study. Twelve percent of the cohort were taking BC risk reduction medication, i.e. tamoxifen, aromatase inhibitors, or raloxifene which is comparable to figures in our clinic [60]. This suggests women will engage in both pharmaceutical and health behaviour risk reduction.

Most women opted to follow the intermittent diet at baseline. However only half of completers were still following the diet at 12 months. This is consistent with previous reports that intermittent diets are not maintained long term [61].

### Strengths

This is one of the few studies to test BC prevention weight loss programmes amongst women attending FHRPCs, and the first to test whether additional CVD and T2D information could increase engagement and behaviour change and weight loss success in this population. Both programmes achieved clinically significant 12-month weight loss (6–7%) which compares favourably with weight loss achieved with low energy diets and weight loss medications [62].

### Limitations

Most of the cohort were recruited from the principal investigator site. Such a pattern is a common in multi-centre studies [63]. This site also had greatest retention. Future multi-centres studies should include more robust support, training and incentives for the participating centres [64]. The retention rate was 55%, which whilst comparable to rates previously reported for 12 month weight loss interventions in other settings [18, 40], makes the trial underpowered and is below an 80% retention so raises concerns about attrition bias and the validity of the trial [65]. Comparable study outcomes between multiple imputation and the conservative baseline observation carried forward analyses however increase confidence in the study findings. An unavoidable cause of drop out was the COVID-19 pandemic, but further drop out in future trials could be reduced with low cost maintained patient contact between 6 and 12 months, potentially using extended phone or text message support and financial incentives to retain participants or the offer of a delayed intervention for the control group.

The cohort was predominately from the white ethnic group, which reflects the low numbers of ethnic minority groups attending UK FHRPCs [66]. Future work should aim to engage women from different backgrounds to FHRPCs and subsequently recruit them to BC prevention programmes. We recruited women across a range of deprivation scores but there was a greater attrition amongst those who were more deprived. This greater attrition aligns to previous reports in the literature, most likely as deprivation is often associated with lower personal agency, i.e. time, resources and education to enable full engagement to a behaviour change interventions [67]. Future interventions should try and minimise this effect to reduce the chances of increasing health inequalities.

### Implications and future research

We have shown that a remotely delivered web and phone weight loss BC prevention programme can be successfully delivered within a UK healthcare setting from a central location to multiple distant centres to FHRPC attendees. The remote programmes were evaluated pre-pandemic and are likely to be more acceptable now. The pandemic has changed public and NHS perceptions for delivering remote programmes online and avoiding the need for hospital visits [68].

An estimated 20% of women in the UK are at moderate or high BC risk [60]. Thus BC prevention programmes need to be accessible with maximal reach across the network of FHRPCs. Additional CVD and T2D risk neither increased nor decreased retention nor weight loss success of the programme.

Further optimisation and decisions around implementation of the intervention will be undertaken based on the qualitative and quantitative process analysis of the present trial, suggesting four main elements that could strengthen an intervention that already shows promise. In particular, greater intensity of intervention during follow-up than the use of automated emails should help with maintenance of weight loss. The use of strategies to help women to better cope with emotional issues that impact on unhealthy eating would be useful. Facilitating peer support amongst participants and a greater focus on physical activity aswell as diet may be warranted. We will aim to strengthen the intervention in consultation with the target population and key stakeholders, i.e. clinicians in high risk clinics and commissioners of these services. This work will also include the development of training materials for dietitians who will be delivering the intervention.

A future large scale RCT across multiple clinics will test whether the BCPP can be implemented across the network of FHRPCs. This trial could test the BCPP against a simpler control group provided with written information only, since the control group herein contained potentially effective elements of the interventions [69].

## Supplementary information

Supplementary figure 1

Supplementary tables

TIDieR Checklist

Consort

## Data Availability

All datasets used and analysed during the current study and the and the trial protocol are available from the corresponding author on reasonable request.
