# Supplementary material for: Randomised controlled trial of breast cancer and multiple disease prevention weight loss programmes vs written advice amongst women attending a breast cancer family history clinic
Source: Br J Cancer. 2023 Feb 25;128(9):1690–700. doi: 10.1038/s41416-023-02207-z (PMC9961304; doi:10.1038/s41416-023-02207-z)
Supplement: Supplementary file 1 — Supplementary figure 1 [file 41416_2023_2207_MOESM1_ESM.docx]

**Supplementary Figure 1 Written advice and the BCPP and MDPP programmes**

| Timeline |  | Health care professional | Modality of contact | Written advice | BCPP | MDPP |
| --- | --- | --- | --- | --- | --- | --- |
|  | **Disease risk information** |  |  |  |  |  |
| Median (range)  5.0 (0 – 27.7) years | Personalised breast cancer risk information had previously been given when women first joined the FHRPC:   - MFT and T&GICFT: 10 year and lifetime percentage estimates derived from the Tyrer-Cuzick model - UHS advised either Moderate lifetime risk (≥17–29.99%) or high lifetime risk (>30%) | Clinician in FHRPC | Face to face / phone /  Letter | ✓ | ✓ | ✓ |
| Time 0 | Recalculation and communication of updated personalised breast cancer risk | Clinician in FHRPC | Phone | ✓ | ✓ | ✓ |
|  | Advice that weight loss of ≥5% and adherence to PA and alcohol recommendations could lead to significant reductions in risk of BC (25%)^1,2^ | Clinician in FHRPC | Phone | ✓ | ✓ | ✓ |
|  | General advice that ≥5% weight loss could reduce their risk of T2D (60%) ^3^and CVD (30%)^4^ | Research dietitian | Phone |  | ✓ | ✓ |
|  | NHS Health Check | Research practitioner | Face to face |  |  | ✓ |
|  | Personalised feedback of:   - CVD risk (10-year and lifetime risk and heart age from QRISK2) ^5^ - T2D risk (QDiabetes and measured HbA1c) (18) | Research dietitian | Phone |  |  | ✓ |
|  | Personalised estimate of change in CVD risk from predicted reductions in blood pressure and total cholesterol (i.e. a 1 mm/Hg reduction in systolic blood pressure per 1% weight loss up to a 10% weight loss ^6^ ^1^ and a 1% reduction in total cholesterol for every 1% weight loss up to a 15% weight loss) ^7^  Personalised estimate of change in Q diabetes risk by entering the target reduced weight in the QDiabetes tool. | Research dietitian | Phone |  |  | ✓ |
|  | NHS Health Check results sent to general practitioner to allow appropriate follow up and clinical management, e.g. checking abnormal result, consideration of medications for raised cholesterol, blood pressure and HbA1c. | General practitioner |  |  |  | ✓ |
|  | **Clinic assessments** |  |  |  |  |  |
| Baseline, 6 and 12 months | Body weight, body composition, waist, hip and blood pressure measurements | Research practitioner | Face to Face | ✓ | ✓ | ✓ |
|  | **Health behaviour advice and behavioural support** |  |  |  |  |  |
| Time 0 | Comprehensive written resources to:   - Follow a weight reducing intermittent (5:2) or daily energy restricted Mediterranean diet including portion guides and recipes. - Meet the physical activity recommendations (150 mins/moderate intensity CV & 40 mins of resistance exercise /week)^8^ - Limit alcohol to <10 units/week due to its effect on weight and independent effects on disease risk |  |  | ✓ | ✓ | ✓ |
| Time 0 | Personalised diet and physical activity advice based on the written resources given. Physical activity advice tailored to participant’s preferences, abilities and co-morbidities, with referral to local services where appropriate^a^ | Research dietitian | Phone |  | ✓ | ✓ |
|  | Tutorial for use of the trial website with self-monitoring and peer support group forum | Research dietitian | Phone |  | ✓ | ✓ |
|  | Referral to NHS smoking cessation, alcohol services if reporting high-risk alcohol intakes with Alcohol Use Disorders Identification Test (AUDIT) scores >8 ^9^ or psychological support if PHQ or GAD ≥15^10,11^ | GP informed of these scores. Participants asked to self-refer to relevant services. | Phone | ✓ | ✓ | ✓ |
| 0 – 6 months | Scheduled review calls at weeks 1, 4 and 8 and 6 months | Research dietitian | Phone |  | ✓ | ✓ |
|  | Personalised e-mails (weeks 2, 3, 5-7, 9-12 then biweekly months 3-6) | Research dietitian | E mail |  | ✓ | ✓ |
|  | Monthly trial newsletter | Automated | E mail | ✓ | ✓ | ✓ |
|  | Self-management using the trial website | Participants | Trial web site |  | ✓ | ✓ |
| 6 - 12 months | Automated monthly email based on trial website entries:   - Positive feedback for records showing weight loss or weight maintenance - Encouraged re-engagement with the programmes if weight had increased by ≥1kg or if no website entries recorded. | Automated | E mail |  | ✓ | ✓ |
|  | Self -management using the trial website | Participants | Trial web site |  | ✓ | ✓ |

a Suitability to follow a home based PA programme was confirmed using the adult pre-physical activity screening system tool^12^, with general practitioner clearance where necessary.

1 Harvie, M. *et al.* Association of gain and loss of weight before and after menopause with risk of postmenopausal breast cancer in the Iowa women's health study. *Cancer Epidemiol.Biomarkers Prev.* **14**, 656-661 (2005).

2 Catsburg, C., Miller, A. B. & Rohan, T. E. Adherence to cancer prevention guidelines and risk of breast cancer. *Int.J.Cancer* (2014).

3 Knowler, W. C. *et al.* 10-year follow-up of diabetes incidence and weight loss in the Diabetes Prevention Program Outcomes Study. *Lancet* **374**, 1677-1686 (2009).

4 Ebrahim, S., Beswick, A., Burke, M. & Davey, S. G. Multiple risk factor interventions for primary prevention of coronary heart disease. *Cochrane.Database Syst.Rev.*, CD001561 (2006).

5 Hippisley-Cox, J. *et al.* Predicting cardiovascular risk in England and Wales: prospective derivation and validation of QRISK2. *Bmj* **336**, 1475-1482 (2008).

6 Jarrett, R. J., Keen, H. & Murrells, T. Changes in blood pressure and body weight over ten years in men selected for glucose intolerance. *J Epidemiol.Community Health* **41**, 145-151 (1987).

7 Aucott, L., Gray, D., Rothnie, H., Thapa, M. & Waweru, C. Effects of lifestyle interventions and long-term weight loss on lipid outcomes - a systematic review. *Obes Rev* **12**, e412-425 (2011).

8 Health., U. D. o. Start Active, Stay Active: A report on physical activity for health from the four home countries' In: Chief Medical Officers. (ed). (2011).

9 Dawson, D. A., Grant, B. F., Stinson, F. S. & Zhou, Y. Effectiveness of the derived Alcohol Use Disorders Identification Test (AUDIT-C) in screening for alcohol use disorders and risk drinking in the US general population. *Alcohol Clin.Exp.Res.* **29**, 844-854 (2005).

10 Kroenke, K., Spitzer, R. L. & Williams, J. B. The PHQ-9: validity of a brief depression severity measure. *J.Gen.Intern.Med.* **16**, 606-613 (2001).

11 Spitzer, R. L., Kroenke, K., Williams, J. B. & LÇôwe, B. A brief measure for assessing generalized anxiety disorder: the GAD-7. *Arch.Intern.Med.* **166**, 1092-1097 (2006).

12 Professor Kevin, N. & Dr Lynda, N. *Pre-exercise Screening: Guide to the Australian adult pre-exercise screening system*. (2011).
