# Supplementary material for: Randomised controlled trial of breast cancer and multiple disease prevention weight loss programmes vs written advice amongst women attending a breast cancer family history clinic
Source: Br J Cancer. 2023 Feb 25;128(9):1690–700. doi: 10.1038/s41416-023-02207-z (PMC9961304; doi:10.1038/s41416-023-02207-z)
Supplement: Supplementary file 2 — Supplementary tables [file 41416_2023_2207_MOESM2_ESM.docx]

**Supplementary Table 1**

**Baseline characteristics of patients who withdraw and complete the study**

| **Variable** | **Missing 12m** n=93 | **Completed 12m** n=117 |
| --- | --- | --- |
| Age- years | 47 (42-51) | 49 (45-52) |
| BMI – kg / m^2^ | 32.0 (28.8-35.8) | 29.9 (27.6-35.8) |
| Anxiety (GAD-7) | 3 (1-7) | 3 (1-6) |
| Depression (PHQ-9) | 4 (2-8) | 3 (2-7) |
| Initial FHC BC risk estimation -TC | 25 (20 -33) | 25 (20 -33) |
| Updated BC risk estimation - TC | 24 (20-30) | 22 (17-30) |
| Difference in initial and updated BC risk estimations | 0 (-6.0,0.0) | -2.7 (-9.0,0.8) |
| Townsend deprivation score | 20 (9-28) | 12 (7.5-21.5) |
| Weight – kg | 87.4  (78.6-96.2) | 80.4  (75.6-96.3) |
| Previous weight loss attempts -n | 5 (2-11) | 4 (2-9) |

Median (IQR)

**Supplementary Table 2: Changes in health behaviours over 12 months**

|  | **Overall cohort** | **Written advice** | **BCPP** | **MDPP** |
| --- | --- | --- | --- | --- |
| **Alcohol – units / week**  Baseline n  3 months – n  6 months – n  12 months    **≥14 units/week n (%)**  Baseline  3 months  6 months  12 months | 205  6 (0-14)  89  3 (0-8)  128  4 (0-11)  115  6 (2.5–12)  51 (24.5%)  10 (11.0%)  21 (16.4%)  16 (16.5%) | 36  8 (1.5-14)  12  7 (3-11)  19  6 (1-9)  18  5(0 -8)  8 (21.6%)  1(8.3%)  2 (10.0%)  1(6.0%) | 85  5 (0-14)  38  2 (0-8)  54  4 (0-10)  48  5 (0 – 8)  19 (22.6%)  3 (7.7%)  8 (14.8%)  7 (16.3%) | 84  6 (0-14)  39  4 (0-10)  55  3 (0-12)  49  10 (5-12)  24(27.6%)  6 (15.4%)  11 (20.4%)  8 (21.6%) |
| **Mediterranean Diet Score**  Baseline – n  3 months – n  6 months – n  12 months- n | 208  6 (5 – 7)  85  9 (7–10)  134  8 (7–10)  114  8 (7-10) | 37  6 (4-7)  4  9 (7-10)  17  8 (7-10)  18  8 (6-9) | 86  6 (5-7)  24  9 (9-10)  49  8 (6-9)  49  8 (6-9) | 85  6 (5-7)  24  9 (7-10)  46  9 (8-10)  47  9 (8-10) |
| **Physical activity -MET/min/ week***  Baseline n  3 months n  6 months – n  12 months- n | 210  231 (99 – 627)  82  382(141 -679)  129  558 (246 – 939)  113  438 (181 – 693) | 37  198 (99-339)  11  332 (90-444)  21  297 (99-396)  16  239 (144-594) | 87  308 (99-717)  35  240 (120-556)  57  273 (132-546)  49  438 (198-798) | 86  239 (99-623)  36  527 (235-749)  51  508 (198-916)  47  438 (198-669) |
| **Current smokers**  Baseline n = 12  12 months n = 5 | 12 210  5/107 | 3/ 37  1 stopped smoking  2 maintained smoking  1 withdrawn | 4/86  1 had reduced smoking  3 withdrawn | 5/87  2 maintained smoking  3 withdrawn |

Median (25- 75^th^ centile) Mediterranean diet score- maximum score 12

*moderate intensity walking = 3.3 METS /minute

**Supplementary Table 3: Baseline cardiovascular and diabetes risk markers and estimated risks in the MDPP group (n = 87)**

|  | Median (95% CI) | Numbers in the risk categories n (%) |
| --- | --- | --- |
| Age - years | 48 (43- 52) |  |
| Heart age - years | 48 (42- 52) | Lower than age: 36 (41%), Equal to age 34 (39%), Higher than age: 17 (20%) |
| Total cholesterol-  mmol/L (n = 86) | 4.6 (4.0-5.2) | Low: 56 (65%), Sub-optimal: 21 (24%), High: 9 (11%) |
| Systolic blood pressure- mmHg  (n = 86) | 129 (120 – 140) | Low: 45 (52%), Sub-optimal: 19 (22%), High: 22 (26%) |
| CVD QRISK2  10 year-%  Lifetime-% | 1.5 (1 – 2.7)  20 (18 – 26) | > 25%: 26 (30%) |
| HbA1c- mmol/mol | 34.0  (33.3 – 34.7%) | Prediabetes ≥42: 1 (1%) |
| Qdiabetes risk  10 year | 4.3 (2 – 12) | Low: 48 (55%), Sub-optimal: 15 (17%), High: 24 (28%)[1] |

Definitions of risk categories Low, Sub – optimal , High [2]

Systolic blood pressure – mm/ hg, < or=129, 130- 139, >or=140

Total cholesterol / mmol/ < 5 5- 5.99 >or= 6

Qdiabetes risk 10 year[1], <or=5.6 5.6-10.5 >10.5

**Supplementary Table 4a Fidelity of delivery of the BCPP and MDPP**

| Elements of the programmes | Participants receiving or engaging with elements of the programme n (%) | | | |
| --- | --- | --- | --- | --- |
|  | BCPP | | MDPP | |
|  | % of women received of women still in the trial | % of women received of women recruited | % of women received of women still in the trial | % of women received of women recruited |
| Initial personalised diet and physical activity advice  Received n (%)  Dietitian time (mins) | 60 (50 – 67) | 85/86 (98%) | 60 (50 – 67) | 86/87 (99%) |
| Dietitian review calls at week 1, 4 and 8 and 6 months  Received n (%)  Dietitian time (mins) | 100 (75-100) %  75 (45–100) | 75 (50-100) % | 75 (75-100) %  80 (48 – 110) | 75 (75-100) % |
| Dietitian e-mails  Sent n (%)  Dietitian time (mins) | 79 (71– 93) %  180 (123– 255) | 79 (64 – 93) % | 79 (64-86) % | 79 (64 – 83) %  195 (130 – 250) |
| Extra dietitian time | 1 participant  (80 mins) |  | 4 participants  (25-90 mins) |  |
| Web site use^*^ |  |  |  |  |
| Used between baseline- 3 months | 81 (100%) | 81 (94%) | 81 (98%) | 81 (93%) |
| Used between 3-6 months | 62 (97%) | 62 (72%) | 62 (94%) | 62 (72%) |
| Used between 6-9 months | ND | 54 (63%) | ND | 49 (56%) |
| Used between 9-12 months | 44 (81%) | 44 (51%) | 35 (70%) | 35 (40%) |
| Received automated e-mail between 9 – 12 months for weight regain of > 1 Kg | 21/51 (41%) |  | 17/50 (34%) |  |
| Number of website entries per participant across the programme | 247 (76 – 603)^a^ |  | 255 (72- 545) ^a^ |  |
| Engagement with peer support forum n (%)  Initial and final date of use  Number of posts/participant    Posts seeking information  Posts seeking support/ peer input | 8 (9%)  1-8 days  1-2  7  5 |  | 13 (15%)  1-155 days  1-4  6  16 |  |

| **Supplementary Table 4b**  Diet choices and NHS referrals for women in the two programmes | | |
| --- | --- | --- |
|  | BCPP | MDPP |
| Diet choice at start of the programme n (%)  Intermittent (5:2)  Daily Mediterranean  Other/ missing | 77 (90%)  7 (8%)  0(0%) 2 (2%) | 75(86%)  8 (9%)  3 (2%) 1 (1%) |
| Diet reported following at 6 months  Intermittent (5:2)  Daily Mediterranean  Other/ missing | 29 (45%)  6 (9%)  2 (3%) 37 (40%) | 31 (47%)  12 (18%)  6 (9%) 27(41%) |
| Diet reported following at 12 months  Intermittent (5:2)  Daily Mediterranean  Other/ missing | 23 (47%)  16 (33%)  7 (14%) 3(6%) | 26 (54%)  15 (31%)  4 (8%) 12 (25%) |
| Referrals to NHS services:  Physical activity  Psychology**  Alcohol services ***  Smoking cessation | 11 (12%)  1 (1%)  1 (**1**%) / out of 3  1 out of 4 smokers | 10 (12%)  4(5%)  1 (1%) out of 4  3 out of 5 smokers |

a mean (95% CI) median ((IQR)

*Used web-site at least once during this period

**Participant met criteria for referral for psychology support (> or=15 General Anxiety Disorder ^2^or (> or=15 Patient Health Questionnaire) ^3^

***Patient encouraged to contact alcohol support services if Audit score ≥16 or ≥35 units/week) ^1^

**Supplementary references**

1. Dawson, D. A., Grant, B. F., Stinson, F. S. & Zhou, Y. Effectiveness of the derived Alcohol Use Disorders Identification Test (AUDIT-C) in screening for alcohol use disorders and risk drinking in the US general population. *Alcohol Clin.Exp.Res.* **29**, 844-854 (2005).
2. Spitzer, R. L., Kroenke, K., Williams, J. B. & Lowe, B. A brief measure for assessing generalized anxiety disorder: the GAD-7. *Arch.Intern.Med.* **166**, 1092-1097 (2006).
3. Kroenke, K., Spitzer, R. L. & Williams, J. B. The PHQ-9: validity of a brief depression severity measure. *J.Gen.Intern.Med.* **16**, 606-613 (2001).
